# Animal signals and emotion in music: coordinating affect across groups

**DOI:** 10.3389/fpsyg.2013.00990

**Published:** 2013-12-25

**Authors:** Gregory A. Bryant

**Affiliations:** Department of Communication, Center for Behavior, Evolution, and Culture, University of California at Los AngelesLos Angeles, CA, USA

**Keywords:** emotion in music, arousal, nonlinearities, music distortion, coalition signaling

## Abstract

Researchers studying the emotional impact of music have not traditionally been concerned with the principled relationship between form and function in evolved animal signals. The acoustic structure of musical forms is related in important ways to emotion perception, and thus research on non-human animal vocalizations is relevant for understanding emotion in music. Musical behavior occurs in cultural contexts that include many other coordinated activities which mark group identity, and can allow people to communicate within and between social alliances. The emotional impact of music might be best understood as a proximate mechanism serving an ultimately social function. Recent work reveals intimate connections between properties of certain animal signals and evocative aspects of human music, including (1) examinations of the role of nonlinearities (e.g., broadband noise) in non-human animal vocalizations, and the analogous production and perception of these features in human music, and (2) an analysis of group musical performances and possible relationships to non-human animal chorusing and emotional contagion effects. Communicative features in music are likely due primarily to evolutionary by-products of phylogenetically older, but still intact communication systems. But in some cases, such as the coordinated rhythmic sounds produced by groups of musicians, our appreciation and emotional engagement might be driven by an adaptive social signaling system. Future empirical work should examine human musical behavior through the comparative lens of behavioral ecology and an adaptationist cognitive science. By this view, particular coordinated sound combinations generated by musicians exploit evolved perceptual response biases – many shared across species – and proliferate through cultural evolutionary processes.

## INTRODUCTION

Musical sounds can evoke powerful emotions in people, both as listeners and performers. A central problem for researchers examining music and emotion is to draw clear causal relationships between affective acoustic features in music and the associated responses in listeners. Behavioral ecologists have long studied emotional communication in non-human animals, and one guiding principle in this research is that the physical forms of evolved signals are shaped by their respective communicative functions (Morton, 1977; Owren and Rendall, 2001). Signals evolve as part of signaling system – that is, the production of a signal is necessarily tied to a systematic response by target listeners. This basic fact of animal signaling leads us to an inescapable conclusion regarding music and emotion: the physical structure of musical forms must be related in important ways to people’s perceptions and behavioral responses to music. The complex question thus arises: does music, in any way, constitute a signal that is shaped by selection to affect listeners’ behavior and potentially convey adaptive information to conspecifics (i.e., members of the same species)? Alternatively, perhaps music is a by-product of a variety of cognitive and behavioral phenomena. In any case, comparative analyses examining acoustic signals in non-human animals can shed light on musical behaviors in people.

Here I will describe research that explores the perception of arousal in music from a comparative perspective, and frame this work theoretically as the exploration of one important proximate mechanism (i.e., an immediate causal process) among many underlying our special attention and attraction to affective properties in musical sound. Music is a cultural product that often exploits pre-existing perceptual sensitivities originally evolved for a variety of auditory functions including navigating sonic environments as well as communication. Cultural evolution has led to increasingly complex, cumulative musical developments through a sensory exploitation process. I suggest that humans have evolved an adaptive means to signal relevant information about coalitions and collective affect within and between social groups. This is accomplished through the incorporation of elaborate tonal and atonal sound, combined with the development of coordinated performance afforded by rhythmic entrainment abilities.

A key issue for understanding the nature of music is to explain why it is emotionally evocative. Darwin (1872) famously described many affective signals in humans and non-human animals, and biologists have since come to understand animal emotional expressions not as cost-free reflections of internal states, but rather as strategic signals that have evolved to alter the behavior of target organisms in systematic ways (Maynard Smith and Harper, 2003). Receivers have evolved response biases that allow them to react adaptively to these signals resulting in co-evolutionary processes shaping animal communication systems (Krebs and Dawkins, 1984). Many scholars have noted the clear connections between human music and emotional vocalizations (Juslin and Laukka, 2003), as well as the connections between human and animal vocalizations (Owren et al., 2011). Snowdon and Teie (2013) recently outlined a theory of the emotional origins of music from a comparative perspective. But researchers examining emotion in music do not typically draw explicit connections to animal vocal behavior.

## FORM AND FUNCTION IN ANIMAL SIGNALS

Recently there has been an increased focus on the form–function relationship between acoustic structure in animal signals and their communicative purposes. The principle of form and function has been indispensable in the study of, for example, functional morphology, but is also crucial for understanding animal signaling. Morton (1977) in his classic paper described the convergent evolution of specific structural features in animal signals based on the behavioral communicative context, and the motivations of senders. Low, broadband (i.e., wide frequency range) sounds are often honestly tied to body size and hostile intent, and can induce fear in receivers. Conversely, high pitched tonal sounds are related to appeasement, and are often produced to reduce fear in listeners. These motivational–structural (MS) rules apply widely across many species and have provided an evolutionary basis for studying the acoustic structure of animal signals (see Briefer, 2012 for a recent review). MS rules illustrate nicely how sound is often much more important than semantics in animals signals. Owren and Rendall (2001) described researchers’ frequent reliance on linguistic concepts in understanding primate vocalizations. Animal signals have often been studied as potentially containing “meaning” with referential specificity. An alternative approach is to examine patterns of responses to closely measured non-referential acoustic features of signals. Many signals can affect perceivers in beneficial ways that that do not require the activation of mental representations, analogs to “words,” or the encoding of complex concepts. Owren and Rendall (2001) encouraged researchers to rule out simple routes of communication before invoking necessarily more complex cognitive abilities that would be required of the signaling organism. That is not to say that complex meanings are never instantiated in non-human animal signals, but that we should not begin with that assumption.

So how do specific acoustic parameters in vocal signals underlie the communicative purposes for which they are deployed? Consider the interactive affordances of the acoustic-startle reflex. Many animal calls consist of loud bursts of acoustic energy with rapid onsets, loudness variation, and nonlinear spectral characteristics that often give the signals a harsh or noisy sound quality. These features serve to get the attention of a target audience, and can effectively interrupt motor activity. The direct effect of this kind of sound on the mammalian nervous system is a function that has been phylogenetically conserved across many taxa. Humans rely on this reflexive principle in vocal behaviors such as infant-directed (ID) speech, crying, pain shrieks, and screams of terror. For instance, in the case of ID speech, prohibitive utterances across cultures contain similar acoustic features – including fast rise times in amplitude, lowered pitch (compared to other ID utterances), and small repertoires (e.g., No! No! No!; Fernald, 1992). These directed vocalizations are often produced in contexts where caretakers want to quickly interrupt a behavior, and must do so without the benefit of grammatical language.

In studies examining the recognition of speaker intent across disparate cultures, subjects are quite able to identify prohibitive intentions of mothers speaking to infants, and other adults as well (Bryant and Barrett, 2007; Bryant et al., 2012). This ability is not a function of understanding the words, but instead due to the acoustic properties of the vocalizations (Cosmides, 1983; Bryant and Barrett, 2008). In the case of ID prohibitives, proximate arousal in senders contributes to the generation of particular kinds of sound features, including rapid amplitude increases and lowered pitch for the authoritative stance. People, including infants, respond in predictable ways to high arousal sounds, such as stopping their motor activity and re-orienting their attention to the sound source. Research with animal trainers also reveals the systematic relationships between specific communicative forms and desired outcomes in animals such as sheep, horses, and dogs (McConnell, 1991). Vocal commands to initiate motor activity in a variety of species typically contain multiple short and repeated broadband calls, while signals intended to inhibit behavior tend to be longer and more tonal. McConnell (1991) also draws an explicit connection to music and cites several older studies from the 1930s showing the above characteristics in music correlating with physiological changes in human listeners. Short repeating rising notes are associated with increased physiological responses such as pulse rate and blood pressure, while longer, slower musical pieces have the opposite effects.

Research has shown that non-human animals respond predictably to musical stimuli, if the music is based on affective calls of their species. Snowdon and Teie (2010) created synthesized musical excerpts that were based on acoustic features of cotton-top tamarin affiliation and threat signals, and they played these compositions, as well as music made for humans, to adult tamarins. Musical stimuli based on threat calls resulted in increased movement, and huddling behavior shortly after exposure. Conversely, the tamarins reacted to affiliation-based music with calming behavior and reduced movement. There was little response to human music, except some reduced movement in response to human threat-based music, suggesting that species-specific characteristics were crucial in eliciting predictable reactions. Because the stimuli did not contain actual tamarin vocalizations, the responses were likely due to structural features of their vocal repertoire, and not merely the result of conditioning. The acoustic structure in the music clearly triggered tamarin perceptual systems designed for perceiving conspecific vocalizations, but importantly, this work demonstrates how acoustic forms can be readily transposed into stimuli we would consider musical, and that it can be affective for non-human listeners. There is some evidence that human music can have effects on non-human animals. Akiyama and Sutoo (2011) found that exposure to recordings of Mozart reduced blood pressure in spontaneously hypertensive rats, and the effect was driven by relatively high frequencies (4 k–16 kHz), an optimal range for rat hearing sensitivity. The authors proposed that the blood pressure reduction was a result of accelerated calcium-dependent dopamine synthesis. These data again show the importance of species-specific response biases in examinations of the effects of musical stimuli on humans and non-humans alike.

Universal form and function relationships are due to the fact that emotional communication systems in animals are evolutionarily conserved (Owren et al., 2011; Zimmermann et al., 2013), and recent work examining the perception of non-human animal affective vocalizations by humans shows that even when people cannot accurately recognize the affect in an animal vocal expression, brain structures react differentially as a function of the emotional valence in the vocalizations. Belin et al. (2008) found that judges could not reliably judge rhesus monkey or cat vocalizations on a positive–negative scale, but still had varying activation in right ventrolateral orbitofrontal cortex (OFC) in response to the recorded vocalizations. There was also greater overall activation for negative affect in the vocal samples, whether produced by human or non-human animals. Other research shows that experience also matters when humans can accurately judge affect in non-human vocal signals. Trained pig ethologists were more accurate than naïve students at classifying the behavioral context of domestic pig vocalizations, and caretakers also systematically judged intensity features as being lower overall (Tallet et al., 2010). Chartrand et al. (2007) found that bird experts had unique brain responses (using EEG) to birdsong than naïve listeners, but the difference extended to environmental sounds and voices as well suggesting that expertise in one domain of auditory processing can affect how people hear sounds in other ways.

### SOUND OF AROUSAL

**FIGURE 1:**
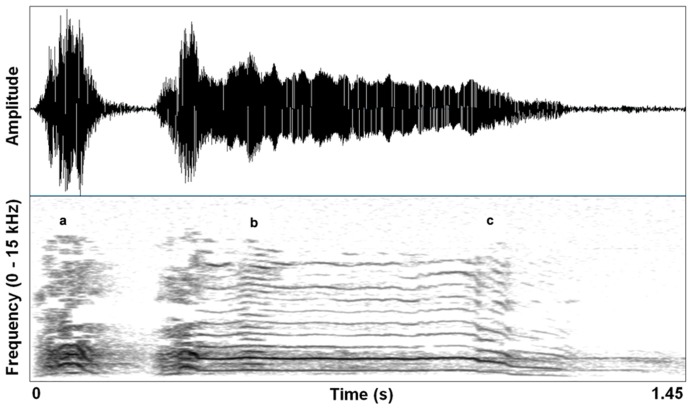
**Waveform and spectrogram (FFT method, window length – 0.005 s, Gaussian window shape, dynamic range – 50 dB) of a single coyote call (*Canis latrans*).** Three nonlinear acoustic features are noted by (a) deterministic chaos, (b) subharmonics, and (c) downward pitch shift. Recording taken from the Macaulay Library collection of the Cornell Lab of Ornithology (ML 125888). Recorded to DAT by Sara Waller, November 2002, California, USA.

Excitement in mammals is often characterized by physiological activation that prepares the animal for immediate action. An emotional state characterized by heightened arousal occurs in context-specific ways, but often motivates vocal communication shaped by selection to affect others’ behavior in an urgent manner. Animals produce pain shrieks, alarm calls, and urgent contact calls, each demanding particular responses perceptually and behaviorally. Specifically in vocalizations, the physiology of high arousal results in increased activation of upper body musculature (including vocal motor systems and respiration) that can cause increased subglottal air pressure and heightened muscle tension. Consequently, vocal folds can vibrate at their natural limit, generating sound waves that reach their maximum amplitude given particular laryngeal and supralaryngeal structural constraints. This saturating nonlinearity (e.g., deterministic chaos) correlates perceptually with a harsh, noisy sound – a sound that effectively penetrates noisy environments, and is hard for listeners to habituate to. **Figure 1** shows a single coyote (*Canis latrans*) contact call that contains subtle deterministic chaos, subharmonics, and a downward pitch shift.

Nonlinearities can be adaptive features of conspicuous signals that require a quick response or certain attention (Fitch et al., 2002). As is the case with many acoustic features of emotional vocalizations, the sound of arousal in scared or excited animals has been conserved across numerous mammal species (Mende et al., 1990; Blumstein et al., 2008; Blumstein and Récapet, 2009; Zimmermann et al., 2013). Researchers examining how noisy features manifest in particular communicative contexts have found that results are not always predictable (e.g., Slaughter et al., 2013) but responses to noisy vocalizations are typically consistent with the idea that these sounds invoke fear in listeners and prepare them for a quick response. Accurate recognition of high arousal in a vocalizer can provide valuable cues concerning threats in the immediate environment, predicting events such as an imminent attack by a conspecific, or an external danger like the approach of a predator. Signaling behavior can evolve from these cues when senders and receivers mutually benefit from the communicative interaction (Maynard Smith and Harper, 2003), and behavioral features often become ritualized in a co-evolutionary process of production enhancement and perceptual sensitivity (Krebs and Dawkins, 1984).

The sound of arousal example provides a very clear logic for why specific sound features (i.e., forms) are associated with systematic emotional reactions and likely subsequent behavioral responses (i.e., functions). Audio engineers and musicians have exploited the sound of arousal in music, and as a result, instrumentation and performances across a variety of music genres seem well-suited to invoke arousal in listeners including inducing fear, excitement, anger, and exhilaration. For the same reasons people watch horror films, ride roller coasters, or surprise each other for amusement, particular sounds in music are interesting and sometimes exciting.

## IS MUSIC SPECIAL?

A complete explanation of the sound features of music is most likely going to be developed from an adaptationist cognitive science informed by a cultural evolutionary framework. The perception and appeal of music is currently best characterized as the co-occurring activation of a collection of by-product perceptual and judgment processes (McDermott, 2009). Pinker (1997) famously described music as “auditory cheesecake” – the theory to beat when proposing adaptive functions for music. It is clear that many systems designed to solve adaptive auditory problems faced recurrently by mammalian species are triggered by phenomena most people would call music. That is, the melodic and rhythmic properties of “musical” sounds satisfy input conditions in a variety of auditory processing mechanisms. Auditory scene analysis research has examined in great detail many fundamental sound perceptual processes and how they relate to navigating the sonic environment (Bregman, 1990). We can segregate sound streams, locate sound sources, and categorize sounds efficiently – abilities that clearly contribute to our perception of music.

Musical forms affect the full range of human emotions. I will focus on the sound of arousal, which often induces fear, as one good example of how a specific vocal phenomenon can manifest itself in music and be perpetuated culturally. This is not intended to explain other emotional phenomena in music, although I would certainly expect similar principles to apply widely across the emotion spectrum. Theories such as these, however, do not fully explain the appeal of Mozart or Bach, for example. Formal accounts of musical structure have laid out in rich detail the hierarchical patterning in tonal organization (e.g., Lerdahl and Jackendoff, 1983), so a complete account of the nature of music must incorporate connections with other aspects of our cognition beyond emotional vocalizations. Snowdon and Teie (2013) proposed four categories of elements to explain the various factors contributing to music. The first two categories involve the development of auditory perception and sensitivity to vocal emotion information. But in the other two categories they point to elements such as melody, harmony, counterpoint, and syntax that are fundamental to the complexity and beauty in music (see also Patel, 2008).

### SPEECH AND MUSIC

Speech is often cited as an important domain contributing to music perception. Speech communication in people has likely resulted in many refinements of phylogenetically older vocal production and perception abilities shared with many non-human animals (Owren et al., 2011). Models of efficient coding of sound also suggest that any specialized auditory processes for speech could be achieved by integrating auditory filtering strategies shared by all mammalian species (Lewicki, 2002). Human hearing sensitivity, however, appears particularly well-attuned to the frequency range of normal speech (Moore, 2008) just as all vocalizing species’ auditory abilities are adapted to conspecific vocalization characteristics. Based on modeling work examining potential filtering strategies of peripheral auditory systems, Lewicki (2002) proposed that the representational coding of speech could be effectively instantiated using schemes specialized for broadband environmental sounds combined with schemes for encoding narrowband (i.e., tonal) animal vocalizations. That is, evolutionarily conserved auditory processes might have constrained speech production mechanisms such that speech sounds fell into frequency and temporal ranges exploiting prelinguistic perceptual sensitivities.

Speech perception is quite robust in normal speakers even in cases where high degradation or interruption is occurring (e.g., Miller and Licklider, 1950), and the temporal rate at which speech can be reliably understood far exceeds the production capability of the most efficient speakers (Goldman-Eisler, 1968). These facts hint at perceptual specialization. But a good deal of our speech processing ability is likely due to auditory abilities widely shared across mammals (Moore, 2008). Cognitive neuroscience research has shown repeatedly that music and speech share brain resources indicating that speech perception systems accept music as input (for recent reviews see Arbib, 2013), though evidence exists for separate processing as well (Zatorre et al., 2002; Peretz and Coltheart, 2003; Schmithorst, 2005). The relationship between speech and music is certainly more than a coincidence. Amplitude peaks in the normalized speech spectrum correspond well to musical intervals of the chromatic scale, and consonance rankings (Schwartz et al., 2003). Many parallels also exist between music and speech development (McMullen and Saffran, 2004).

The physical properties of the sounds are not the only dimensions that link speech and music. The structure of various sound sequences also seems to activate the same underlying cognitive machinery. Research examining rule learning of auditory stimuli demonstrates the close connection between perceiving speech and music. Marcus et al. (2007) found that infants could learn simple rules (e.g., ABA) in consonant–vowel (CV) sequences, and the learning can apply to non-speech stimuli such as musical tones or non-human animal sounds. However, extracting rules from sequences of non-speech stimuli was facilitated by first learning the rules with speech, suggesting that the proper domain (see below) of rule learning in sound sequences is speech, but musical tones and other sounds satisfy the input conditions of the rule learning system once the system is calibrated by spoken syllables. Studies exploring the acquisition of conditional relations between non-adjacent entities in speech or melodic sequences show similar patterns (Creel et al., 2004; Newport and Aslin, 2004).

A good deal of music perception is likely due to the activity of speech processing mechanisms, but perception is only half of the system. We should be concerned with how production and perception systems evolved together. There are clear adaptations in place underlying breathing processes in speech production and laryngeal and articulator control (MacLarnon and Hewitt, 1999). Moreover, we have fine cortical control over pitch, loudness, and spectral dynamics (Levelt, 1989). These production systems, as a rule of animal signaling, must have complementary adaptive response patterns in listeners. Many perceptual biases were in place before articulated speech evolved, such as the categorical perception of continuous sounds (Kuhl, 1987). But other response biases might be new, such as sensitivity to the coordinated isochronic (i.e., steady, pulse-based repetition) rhythms produced by multiple conspecifics. Sperber (1994) made a distinction between the proper domain of a mechanism and its actual domain. Proper domain refers to those specific features that allow a system to solve an adaptive problem. Depending on the nature of the dynamics (i.e., costs and benefits) of the adaptation, systems will vary in how flexible the input conditions are to respond to a stimulus. The actual domain of a system is the range of physical variation in stimuli that will result in a triggering of that mechanism, something that is often a function of context and the evolutionary history of the cognitive trait. In these terms, the actual domain of speech processers presumably includes most music.

Domain specificity in auditory processing can illuminate the nature of people’s preferences for certain sounds, including why certain musical phenomena are so interesting to listeners. But how these preferences manifest themselves as social phenomena remains to be explained. One possibility is that cultural evolutionary processes act on those sound characteristics that people are motivated to produce and hear. For example, rhythmic sound that triggers spatial localization mechanisms could be preferred by listeners, and consequently be subject to positive cultural selection resulting in the feature spreading through musical communities. Other examples include singing patterns that exaggerate the sound of affective voices, or frequency and amplitude modulations that activate systems designed to detect speech sounds. The question becomes, of course, is any sound pattern unique to music?

### CULTURAL TRANSMISSION OF MUSICAL FEATURES

Researchers are starting to explore how listeners’ specific sound preferences can lead to the evolution of higher order structure that can constitute eventual musical forms. MacCallum et al. (2012) created a music engine that generates brief clips of sounds that were judged by listeners – clips that started out quite non-musical. Passages that were preferred in forced-choice trials “reproduced,” that is, were recombined with other preferred passages. This evolutionary process resulted in several higher order structures manifesting as unquestionably musical attributes. For instance, an isochronic beat emerged. Understanding perceptual sensitivities (i.e., solutions to auditory processing adaptive problems) that are relevant in music listening contexts will help explain preference patterns, and evolutionary cultural processes can provide a framework for understanding the proliferation of these sensitivities (Merker, 2006; Claidière et al., 2012). The sound of fear represents one dimension of auditory processing relevant for music which is in place because of conserved signaling incorporating arousal. As a consequence, people are interested in sounds associated with high arousal, and cultural transmission processes perpetuate them.

Consider the form and function of punk rock in western culture. The relevant cultural phenomena for a complete description of any genre of music are highly complex, and not well understood. But we can clearly recognize some basic relationships between the sonic nature of certain genres of music and their behavioral associations in its listeners. Like much music across culture, there is a strong connection between music production and movement in listeners, epitomized by dancing, resulting in a cross-cultural convergence on isochronic beats in music traditions. The tight relationship between musical rhythm perception and associated body movement is apparent in babies as young as seven months (Phillips-Silver and Trainor, 2005). Punk rock is no exception. Early punk is characterized by a return to fundamentals in rock music (Lentini, 2003). It began as a reaction to a variety of cultural factors, and the perceived excesses of ornate progressive music in general. The initial creative ethos was that anybody can do it, and it was more of an expression of attitude than the making of cultural artifacts. In short, it was intense (and sometimes aggressive) in many ways, and whatever one’s interpretation of the cultural underpinnings, the energy is apparent. The music is characterized by fast steady rhythms, overall high amplitude, and noisy sound features in all instruments – attributes that facilitate forceful dancing. But the distortion noise is especially distinct and key for the genre. Of course, many genres of rock use noise – the punk example is just preferred here for many cultural and explanatory reasons, but the same principle applies to many variations of blues and rock music.

Noisy features in rock took a life of their own in the No Wave, post punk, and experimental movements of the 1980s and beyond (e.g., O’Meara, 2013). In rock music, what originally likely arose as a by-product of amplification (i.e., attempting to be loud along with an intense style of playing) soon became conventionalized in ways that are analogous to ritualization in the evolution of animal signals (Krebs and Dawkins, 1984). Particular manifestations of noisy features (forms) were directly related to compositional and performance goals of musicians (functions). Products were developed that harnessed particular kinds of distortion in devices (e.g., effects pedals) that modified the signal path between an instrument and the amplifier. This allowed artists to achieve the desired distortion sounds without having to push amplifiers beyond their natural limit. The use of noise quickly became a focus of a whole family of musical styles, most being avant garde and experimental. Continuing the trend of rejecting aspects of dominant cultural practices, artists could signal their innovation and uniqueness by using this new feature of music in ways that set them apart. The sound affordances of broadband noise provide a powerful means for artists to generate cultural attractors fueled by discontent with mass market music. Moreover, the creative use of distortion and other effects can result in spectrally rich and textured sounds. Cultural evolutionary forces will tap into any feature that allows socially motivated agents to differentially sort based on esthetic phenomena (Sperber, 1996; McElreath et al., 2003). Simple sound quality dimensions like intensity might be excellent predictors of how people are drawn to some genres and not others (Rentfrow et al., 2011). Listeners also often find moderate incongruities (as opposed to great disparities) between established forms and newer variations the most interesting (Mandler, 1982). For example, modern noise rock with extreme distortion that is quite popular today would likely have been considered much more unlistenable in 1960 because it is such a dramatic departure from the accepted sounds for music at the time. But today it is only slightly noisier than its recent predecessors. What gets liked depends on what is liked.

### DISTORTION, AROUSAL, AND MUSIC

**FIGURE 2:**
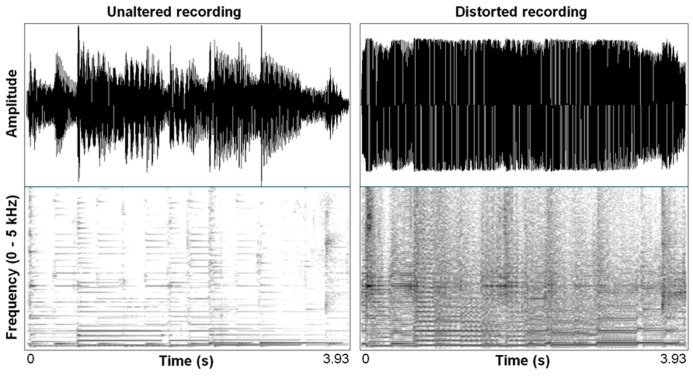
**Waveform and spectrogram (FFT method, window length – 0.005 s., Gaussian window shape, dynamic range – 50 dB) of an acoustic guitar melody unaltered, and distorted using a wave shaping function (Camel Crusher VST Plug-in)**.

**FIGURE 3:**
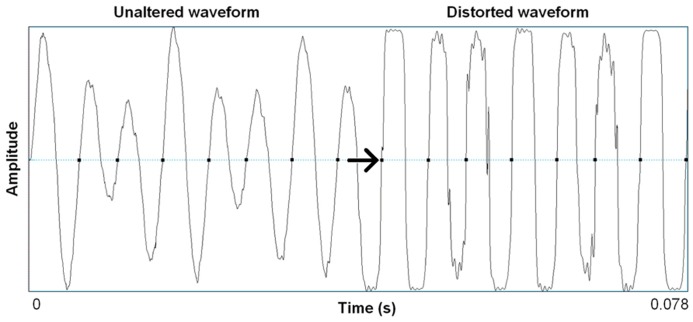
**Waveform segments taken from recording shown in Figure 2 of seven cycles (78 ms) of unaltered acoustic guitar, and the same seven cycles after wave shaping function (Camel Crusher VST Plug-in).** Arrow notes onset of wave shaping.

Distortion effects in contemporary music mimic in important ways the nonlinear characteristics we see in highly aroused animal signals, including human voices. Electronic amplification, including the development of electro-magnetic pick ups in guitars, was arguably the most important technological innovation that led to the cultural evolution of rock music, and the situation afforded an incredible palette of sound-making that is ongoing well over half a century later (Poss, 1998). Just in the same ways that an animal’s vocal system can be “overblown,” so can the physical hardware of amplification systems. Early garage rock music, the precursor to punk rock, was likely the first genre to systematically use this overblown amplification effect on purpose. Specific manipulations of electronic signal pathways were developed that allowed musicians to emulate in music what is an honest feature of a vocalization: high arousal. A basic distortion pedal works as follows. The first process is typically an amplitude gain accompanied by a low-pass filter, pushing the signal toward a saturation point where nonlinear alterations will occur. This saturating nonlinearity is filtered again, resulting in output that becomes a multi-band-passed nonlinearity. **Figure 2** shows the effect of a wave shaping function on a 4 s recording of an acoustic guitar and **Figure 3** shows a 78 ms close-up segment of several cycles of the complex waveform in both unaltered and distorted treatments. Yeh et al. (2008) have used ordinary differential equations (ODEs) to digitally model this analog function suggesting that analog distortion used by musicians closely approximates noisy features in vocalization systems that are also well described by the same mathematics. **Figure 1** shows the spectrogram of a coyote vocalization with subtle nonlinear phenomena that appear quite similar to broadband noises generated by ODEs.

Recently, we produced musical stimuli to examine the role of noise in emotional perceptions of music, and used digital models created for musicians as our noisy source (Blumstein et al., 2012). Twelve 10 s compositions were created that were then manipulated into three different versions: one with added musical distortion noise, one with a rapid frequency shift in the music, and one unaltered control. The manipulations were added at the halfway point in the pieces. These stimuli were played to listeners and they were asked to rate them for arousal and valance. We expected that distortion effects approximating deterministic chaos would cause higher ratings of arousal, and negative valence judgments – the two dimensional description of vocalized fear (Laukka et al., 2005). This is precisely what we found. Subjects also judged rapid pitch shifts up as arousing, but not pitch shifts down. Downward pitch shifts were judged as more negatively valenced which is what we should expect given the acoustic correlates of sadness in voices (Scherer, 1986). Surprisingly, previous work had not explored the role of distortion in affective judgments of music, but an animal model of auditory sensitivity afforded a clear prediction which was confirmed.

We were interested in how these effects occurred in the context of film. Previous work had found that horror soundtracks contained nonlinearities at a much higher rate than other film genres (Blumstein et al., 2010). Film soundtrack composers were exploiting people’s sensitivity to noisy features in their efforts to scare or otherwise excite their viewers. Of course, for the most part the direct connection is not consciously made between the ecology of fear screams in animals and the induction of fear in a human audience. But composers and music listeners have an intuitive sense of what sounds are associated with what emotions, and this intuition is rooted in our implicit understanding of form and function in nature – a principle that is strongly reinforced by cultural processes bringing these sounds to us repeatedly generation after generation.

But would sound features alone be sufficient to invoke fear even in the context of an emotionally benign film sequence? We created simple 10-s videos of people engaged in emotionally neutral actions, such as reading a paper, or drinking a cup of coffee. The videos were edited so that the key “action” happened at the exact midpoint, the same time that our nonlinear features in the music clips occurred. Subjects viewed these videos paired with the same music as described above, and we found something interesting. Judgments of arousal were no longer affected by the nonlinear features in the music clips when viewed in the context of a benign action, but the negative valence remained. Clearly, decision processes used in judgments of affect in multimodal stimuli will integrate these perceptual dimensions. One obvious possibility for our result is that the visual information essentially trumped the auditory information when assessing urgency, but the emotional quality of a situation was still shaped by what people heard. Future research should explore how consistent fearful information is processed, and we should expect that auditory nonlinearities will enhance a fear effect as evidenced by the successful pairing of scary sounds and sights in movies. Currently, we are examining psychophysiological responses to nonlinearities, with the expectation that even when judges do not explicitly report greater arousal while hearing nonlinear musical features in certain contexts, there will be measurable autonomic reactions, similar to how brain (OFC) responses to non-human animal voices do not correspond to people’s judgments (Belin et al., 2008).

As mentioned earlier, nonlinear characteristics in music represent one dimension in sound processing that plays a role in music perception and enjoyment. Our sensitivity to such features is rooted in a highly conserved mammalian vocal signaling system. I argue that much of what makes music enjoyable can be explained similarly. But one aspect of music that is not well explained as a by-product is the conspicuous feature that it is often performed by groups – coordinated action of multiple individuals sharing a common cultural history, generating synchronized sounds in a context of ritualized group activity.

## MUSIC AS COALITION SIGNALING

Humans are animals – animals with culture, language, and a particular set of cognitive adaptations designed to interface with a complex social network of sophisticated conspecifics. Pinker (2010) called this the “cognitive niche” taking after ideas earlier proposed by Tooby and DeVore (1987). Information networks and social ecologies have co-evolved with information processors, and thus, a form–fit relationship exists between the cognitive processes in the human mind and the culturally evolved environments for social information. Humans cooperate extensively – in an extreme way when viewed zoologically – and we have many reliably developing cognitive mechanisms designed to solve problems associated with elaborate social knowledge (Barrett et al., 2010). Because many of the adaptive problems associated with extreme sociality involve communicating intentions to cooperate as well as recognizing cues of potential defection in conspecifics, we should expect a variety of abilities that facilitate effective signaling between cooperative agents.

Many species, ranging from primates, to birds, to canines, engage in coordinated signaling. By chorusing together, groups can generate a signal that honestly communicates their numbers, and many other properties of their health and stature. Chorusing sometimes involves the ability to rhythmically coordinate signal production. When two signaling systems synchronize their periodic output (i.e., enter a phase relationship), it can be described as entrainment – an ability that is phylogenetically old, and evolutionarily widespread (Schachner et al., 2009; Phillips-Silver et al., 2010). Fitch (2012) described the paradox of rhythm, which is the puzzle of why periodic phenomena are so ubiquitous in nature, but overt rhythmic ability in animals is so exceedingly rare. The answer, Fitch argued, lies in how we conceptualize rhythm in the first place. When we consider the component abilities that contribute to our capacity for rhythmic entrainment, the complexity in the neurocomputational underpinnings makes the capacity much less paradoxical, and instead understandably rare.

The basic ability to coordinate behavior with an external stimulus requires at a minimum three capabilities: detecting rhythmic signals, generating rhythms through motor action, and integrating sensory information with motor output (Phillips-Silver et al., 2010; Fitch, 2012). Phillips-Silver et al. (2010) described the ecology of entrainment, and the assortment of its manifestations in nature. While many species have variations of these abilities, only humans seem to have a prepared learning system designed to govern coordinated action of a rhythmic nature. The ability to entrain with others develops early, and is greatly facilitated by interactions with other social agents, but not mechanized rhythmic producers, or auditory stimuli alone (Kirschner and Tomasello, 2009). Young infants reliably develop beat induction quite early (Winkler et al., 2009) and have also been shown to engage rhythmically with music stimuli without the participation of social agents, which is associated with positive affect (Zentner and Eerola, 2010). Most rhythmic ability demonstrated by human infants has never been replicated in any other adult primate. Even with explicit training, a grown chimpanzee cannot entrain their rhythmic production with another agent, let alone another chimpanzee. African apes, including chimps and gorillas, will drum alone, and this behavior is likely to be homologous with human drumming (Fitch, 2006), suggesting that coordinated (as opposed to solo) rhythmic production evolved after the split with the last common ancestor. So what is it about the hominin line that allowed for our unique evolutionary trajectory in the domain of coordinated action?

There are other species that have the ability to entrain their behavior to rhythmic stimuli and other agents. Birds that engage in vocal mimicry, such as the sulfur-crested cockatoo (*Cacatua galerita*) have been shown to be capable of highly coordinated responses to music and rhythmic images, and will even attempt to ignore behaviors around them produced by agents who are not in synch with the stimulus to which they are coordinated (Patel et al., 2009). African gray parrots (*Psittacus erithacus*) also have this ability (Schachner et al., 2009). Recently, Cook et al. (2013) found motor entrainment in a California sea lion (*Zalophus californianus*), an animal that does not have vocal mimicry skills, suggesting that the ability either does not require vocal mimicry mechanisms, or the behavior can emerge through multiple motor control pathways. Fitch (2012) pointed out that examining these analogous behaviors can quite possibly elucidate human adaptations for entrainment, but he did not address the larger question of why humans might possess entrainment abilities uniquely across all terrestrial mammals.

Hagen and Bryant (2003) proposed that music and dance constitute a coalition signaling system. Signals of coalition strength might have evolved from territorial displays seen in other primates, including chimpanzees (Hagen and Hammerstein, 2009). The ideal signal of coalition quality should be easily and rapidly decoded by a target audience, and only plausibly generated by stable coalitions able to engage in complex, coordinated action. A coordinated performance affords an opportunity to signal honest information about time investments with fellow performers, individual skills related to practice time investment, and creative ability indicating cognitive competence. In short, individuals can signal about themselves (which could be subject to sexual selection), and the group can signal about their quality as well. To test these ideas, original music was recorded, and versions were made that contained different kinds of performance errors (Hagen and Bryant, 2003). As expected, the composition with introduced errors that disrupted the synchrony between the performers was judged by listeners as lower in music quality. We also asked the listeners to judge the relationships between the performers, including questions about how long they have known each other, and whether they liked each other. Listeners’ judgments of the coalition quality between the performers were a function of the music quality judgments – the lower they rated the music quality, the worse coalition they perceived between the musicians.

The ethnographic record clearly reveals the importance of music and dance displays to traditional societies throughout history (Hagen and Bryant, 2003). Initial meetings where groups introduce one another to their cultures, including these coordinated displays, can have crucial adaptive significance in the context of cooperation and conflict. The potential for selection on such display behaviors is clear, as is the important interface with cultural evolutionary processes (McElreath et al., 2003). Cultural traditions that underlie the nature of specific coordinated displays are revealed in contemporary manifestations of the role of music in social identity and early markers of friendship preferences and alliances (Mark, 1998; Giles et al., 2009; Boer et al., 2011). Mark (1998) proposed an ecological theory of music preference suggesting that music can act as a proxy for making judgments about social similarity. According to the theory, musical preferences spread through social network ties unified by principles of social similarity and history. Investment of time in one preference necessarily imposes time constraints on other preferences. Developing a strong esthetic preference, therefore, can honestly signal one’s social affiliation.

Music can also function to increase coalition strength within groups (McNeill, 1995) and this effect has been documented in children. Kirschner and Tomasello (2010) had pairs of 4-year-old children partake in one of two matched play activities that differed only in the participation of a song and dance. The musical condition involved singing to a prerecorded song (with a periodic pulse) while striking a wooden toy with a stick, and walking to the time. The non-musical condition involved only walking together in a similar manner with non-synchronized utterances. Pairs of children who participated together in the musical condition spontaneously helped their partner more in a set-up scenario immediately after the play activity where one needed assistance, and they engaged in more joint problem solving in that set-up as well. Our proximate experiences of pleasure in engaging with other social agents in musical activity might serve to bolster within-group relationships, and provide a motivating force for generating a robust signal of intragroup solidarity that can be detected by out-group members.

Patterns of cultural transmission occur through different channels. Many cultural traits get passed not only vertically from older members of a culture to their offspring, but also horizontally across peers. For instance, children typically will adopt the dialect and accent of their same-aged peers rather than their parents (Chambers, 2002), illustrating how language learning and communicative-pragmatic mechanisms are quite sensitive to the source of its input. Similarly, peers should be an important source of musical taste development if that esthetic is important for social assortment (Selfhout et al., 2009). Variations of forms in any cultural domain will typically cluster around particular attractors, but the nature of the attraction depends on the type of artifact. For instance, artifacts such as tools that have some specific functional use will be selected based largely (though not completely) on physical affordances (e.g., hammers have the properties they have because they have undergone selection for effectiveness in some task), whereas esthetic artifacts tap into perceptual sensitivities that evolved for reasons other than enjoying or using the artifacts. For example, people prefer landscape portrayals with water over those without water because of evolved foraging psychology (Orians and Heerwagen, 1992). As described earlier, music exploits many auditory mechanisms that were designed for adaptive auditory problems like speech processing, sound source localization, or vocal emotion signaling. Physical characteristics of musical artifacts that appealed to people’s perceptual machinery were attractive, and as a result, the motivation to reproduce and experience these sounds repeatedly provides the groundwork for cultural selection.

Many proposals exist describing potential factors that might contribute to the spreading of any kind of cultural product, and theorists debate about the nature of the representations (including whether they need to be conceived as representations at all) and what particular dynamics are most important for the successful transmission of various cultural phenomena (Henrich and Boyd, 2002; McElreath et al., 2003; Claidiere and Sperber, 2007). In the case of music, some aspects seem relatively uncontroversial. For example, the status of an individual composer or a group of individual music makers likely plays an important role in whether musical ideas get perpetuated. A coordinated display by the most prestigious and influential members of a group was likely to be an important factor in whether the musical innovations by these people were learned and perpetuated by the next generation. Subsequent transmission can be facilitated by conformity-based processes. A combination of factors related to the physical properties of the music, the social intentions and status of the producers, and the social network dynamics of the group at large will all interact in the cultural evolution of musical artifacts. McElreath et al. (2003) showed formally that group marking (which in an ancestral environment could quite plausibly have included knowledge of specific musical traditions), can culturally evolve and stabilize if participants preferentially interact in a cooperative way with others who are marked like them, and they acquire the markers (e.g., musical behaviors) of successful individuals. By this formulation, acquired arbitrary musical markers can honestly signal one’s past cooperative behavior beyond the investment to develop the marker, and potentially provide that information to outside observers.

### EMOTIONS AND MUSIC IN GROUPS

There are many possible evolutionary paths for the perpetuation of musical forms, any even the propensity for musical ability in the first place (e.g., Miranda et al., 2003). But how does emotion play into the process? Little research has explored directly the affective impact of group performances aside from the evocative nature of the music itself. The feelings associated with experiencing coordinated action between groups of people might not fit into a traditional categorical view of emotions, and instead may be better categorized as something like profundity or awe (Davies, 2002; Keltner and Haidt, 2003). According to the coalition signaling perspective, elaborate coordinated performances are an honest signal that is causally linked to the group of signalers. This view does not require any specific affective component, at least not in the traditional approach of studies on emotion and music. The affect inducing qualities of music facilitate its function in that the generated product is inherently interesting to listeners and relevant to the context-specific emotional intentions of the participants. The surface features of the signals satisfy input conditions of a variety of perceptual systems (i.e., they act proximately), and cultural processes perpetuate these characteristics because coordinated displays that embody esthetically attractive displays do better than alternatives. But the ultimate explanation addresses how coordinated displays provide valuable information about the group producing it. A form–function approach again can illuminate the nature of the signaling system and how it operates. Musical features such as a predictable isochronic beats and fixed pitches facilitate the coordinated production of multiple individuals and afford a platform for inducing intended affect in listeners. Our perceptual sensitivity to rhythm and pitch, also important for human speech and other auditory adaptations, allow listeners to make fine grained judgments about relationships between performers. We can tell if people have practiced, whether they have skill that requires time, talent, and effort, and whether they have spent time with the other performers.

Hatfield et al. (1994) developed the idea of emotional contagion as an automatic and unconscious tendency of people to align behaviorally as a means to transfer affect across multiple individuals. Contagion effects in groups are likely connected to a variety of non-human animal behaviors. Several primate species seem to experience some version of contagious affect, including quite notably the pant hoots of chimpanzees that could be phylogenetically related to music behavior in humans (Fritz and Koelsch, 2013). While rhythmic entrainment is zoologically rare, other acoustic features can be coordinated in non-human animals signals, a phenomenon Brown (2007) calls contagious heterophony which he believes played a crucial role in the evolution of human music. In the case of people, Spoor and Kelly (2004) proposed that emotions experienced by groups might assist in communicating affect between group members and help build social bonds. Recent work shows that the transmission of emotion across crowds can act like an unconscious cascade (Dezecache et al., 2013), so the utility of a unifying source of affect (e.g., music) is clear. While all of these ideas are likely to be part of the human music puzzle, scholars have neglected to develop the idea of how coordinated musical action might constitute a collective signal to people outside of the action. Many of the claimed benefits of coordinated action, such as increased social cohesion and alignment of affect, might be proximate mechanisms serving ultimate communicative functions. As is common in the social sciences, proximate mechanisms are often treated as ultimate functions, or function is not considered at all.

Evidence is mounting that affect is not necessarily tied to synchronous movement or the benefits associated with it. A variety of studies have shown that positive affect is not needed for successful coordination, and that explicit instruction to coordinate action can result in cooperative interactions without any associated positive emotions being experienced by participants (e.g., Wiltermuth and Heath, 2009). Recent research has demonstrated that strangers playing a prisoner’s dilemma (PD) economic game after a brief conversation were more likely to cooperate with one another as a function of how much they converged in their speech rate (Manson et al., 2013), and this effect occurred independent of positive emotions between conversationalists. Language style matching was also not related to cooperative moves in the PD game, suggesting that coordinated action can impact future interaction behavior without mediating emotions or behavior matching lacking temporal structure.

The role of emotions in group musical performances is not clear, but what is intuitively obvious is that the experience of a group performance is often associated with feelings of exhilaration, and a whole range of emotions. But such emotional experiences are necessarily tied up in the complexities of the social interaction, and the cultural evolutionary phenomena that contribute to the transmission of the musical behavior. Researchers should examine more closely how specific emotions are conjured during group performances: in players, dancers, and audience members alike. Moreover, how much of the impact of the emotional experience is due to the particular structural features of the music, independent of the coordinated behavioral components? In players and listeners, the psychological concept of the “groove” is related to easily achievable sensorimotor coupling and an associated positive emotional experience (Janata et al., 2012), which is consistent with notions of “flow” that underlie a broad range of individual and coordinated behaviors (Csikszentmihalyi, 1990). Flow can be thought of as an experiential pleasure that is derived from certain moderately difficult activities, and it can facilitate the continued motivation to engage in those activities. One study examined flow in piano players, and found that several physiological variables such as blood pressure, facial muscle movements, and heart rate measures were positively correlated with self-reported flow experiences (de Manzano et al., 2010). The psychological constructs of the groove and flow speak to both the motivational mechanisms underlying music, and the high degree of shared processing that many musical and non-musical phenomena share. In many cultures, the concept of music as separate from the social contexts and rituals in which it manifests is non-existent (Fritz and Koelsch, 2013). The western perspective has potentially isolated music as a phenomenon that is often divorced from the broader repertoire of behaviors in which is typically occurs, and this situation might have important consequences for understanding it as an evolved behavior (McDermott, 2009).

## CONCLUSION

Music moves us – emotionally and physically. The physical characteristics of music are often responsible, such as the wailing sound of a guitar that is reminiscent of a human emotional voice, or the solid beat that unconsciously causes us to tap our foot. The reasons music has these effects are related in important ways to the information-processing mechanisms it engages, most of which did not evolve for the purposes of listening to music. Music sounds like voices, or approaching objects, or the sounds of animals. Cognitive processes of attraction, and cultural transmission mechanisms, have cumulatively shaped an enormous variety of genres and innovations that help people define themselves socially. Music is an inherently social phenomenon, a fact often lost on scientists studying its structure and effects. The social nature of music and the complex cultural processes that have led to its important role in most human lives strongly suggests an evolutionary function: signaling social relationships. Evidence of adaptive design is there: people are especially susceptible to the isochronic beats so common across cultures, we are particularly skilled like no other animal in coordinating our action with others in a rhythmic way, and the ability develops early and reliably across cultures. Group performances in music and dance are universal across all known cultures, and they are usually inextricably tied to central cultural traditions.

Several predictions emerge from this theoretical perspective. For example, if listeners are attuned to the effects of practice on well-coordinated musical displays as a proxy for time investment and group solidarity, then manipulations of practice time between a set of musicians should affect subjects’ judgments on a variety of perceptual measures, including measures that do not explicitly ask about the musical performance. Subjects should be able to readily judge coalition quality through music and dance production (Hagen and Bryant, 2003). High resolution analyses of synchrony between performers should be closely associated with listeners’ assessments of social coordination, and this association should be independent of the assessment of any individual performer’s skills. Researchers need to closely examine the developmental trajectory of entrainment abilities and begin to explore children’s ability to infer social relationships based on coordinated displays. Kirschner and Tomasello (2009, 2010) have begun work in this area that I believe will prove to quite fruitful in understanding the nature of group-level social signaling.

The current approach also makes predictions about the culturally evolved sound of music. We should expect musical elements to exploit pre-existing sensory biases, including sensitivity to prosodic signals conveying vocal emotion in humans and non-human animals (Juslin and Laukka, 2003; Blumstein et al., 2012) and sound patterns that facilitate auditory streaming (Bregman, 1990), for example. These characteristics should be stable properties of otherwise variable musical traditions across cultures, and persistent across cultural evolutionary time. One obvious case described earlier is the perpetuation of electronically generated nonlinearities across a broad range of musical styles today that can be traced back to fairly recent technological innovations. In a matter of a few decades, most popular music now includes nonlinear features of one sort or another that only experimental avant-garde music used before. Indeed, sound features present in the vocal emotions of mammalian species are reflected in the most sophisticated instrumentation of modern classical and jazz. Following Snowdon and Teie (2010, 2013) we should also expect to find predictable responses in many non-human animals to musical creations based on the structural features of their emotional vocal signals. The question of why humans have evolved musical behavior, and other social animals have not, can only be answered by understanding the nature of culture itself – no small task.

Comparative analyses provide crucial insights into evolutionary explanations for any behavioral trait in a given species. In the case of human music, there is clear uniqueness, but we recognize traits common across many species that play into the complex behavior (Fitch, 2006). Convergent evolutionary processes lead to structural similarities across diverse taxa, such as the relationships between birdsong and human music (e.g., Marler, 2000; Rothenberg et al., 2013), and while there are possible limitations in what we can learn from such analogies (McDermott and Hauser, 2005), there is certainly value in exploring the possibilities. Many animals signal in unison, or at least simultaneously, for a variety of reasons related to territorial behavior, and mating. These kinds of behaviors might be the most important ones to examine in our effort to identify any adaptive function of human musical activity, as the structural forms and typical manifestations of human music seem particularly well-suited for effective and efficient communication between groups. This is especially interesting considering the fact that music often co-occurs with many other coordinated behaviors such as dancing, and themes in artifacts like clothing and food. Music should be viewed as one component among many across cultures that allows groups to effectively signal their social identity in the service of large scale cooperation and alliance building. The beautiful complexity that emerges stands as a testament to the power of biological and cultural evolution.

## Conflict of Interest Statement

The author declares that the research was conducted in the absence of any commercial or financial relationships that could be construed as a potential conflict of interest.
